# Transcortical versus transsylvian approaches for adult insular glioma resection: a systematic review with subanalysis by Berger–Sanai segmentation

**DOI:** 10.1007/s10143-026-04253-6

**Published:** 2026-04-02

**Authors:** Gustavo Wagner Baratti Rocha, Paulo Gabriel Sacramento de Silva, Gabriela Monteiro Pereira, Enzo Muzi, Victor de Souza Antunes, Bruno Sodré Serra Antunes, Gabriela Luiza Veloso Maia, Luccas Leite da Silva, Marcos Vinícius Calfat Maldaun, Enrico Ghizoni, Cleiton Formentin

**Affiliations:** 1 Faculty of Medical Sciences, Department of Neurology, State University of Campinas, Campinas, Brazil; 2 Medical School, Pontifical Catholic University of Campinas, Campinas, Brazil; 3 School of Medicine São Leopoldo Mandic, Medical School, Campinas, Brazil; 4 Hospital Sírio-Libanês, São Paulo, Brazil

**Keywords:** Insula, Insular glioma, Transcortical, Transsylvian, Berger-sanai

## Abstract

Insular gliomas pose a neurosurgical challenge due to their deep location and proximity to other structures. The transsylvian (TS) and transcortical (TC) approaches remain the main techniques, guided by the Berger–Sanai (BS) classification. This study compares outcomes across BS zones. A systematic literature review, following PRISMA 2020 guidelines, was conducted in PubMed, Embase, Cochrane Library, and Google Scholar (2010–2025), including adult patients undergoing insular glioma resection via TS or TC approaches, stratified by BS zones. Studies reporting Extent of Resection (EOR) and neurological outcomes were eligible. Data quality was assessed and results were synthesized according to tumor location and surgical approach. As only previously published data were analyzed, ethical approval was not required. Fourteen studies (1,225 patients: 990 TC; 235 TS) were included. TC predominated and generally achieved higher EOR, while TS produced similar results in favorable regions, especially BS Zones I–II. Overall, permanent deficits were uncommon. Zone I consistently allowed maximal safe resection; Zone II showed a trade-off between EOR and ischemic risk, with mixed evidence and a slight advantage for TS. Zones III–IV were sparsely reported, with data favoring TC for deeper control. Both approaches permit safe resection with low rates of permanent morbidity. TC remains the dominant strategy and often attains superior EOR, whereas TS is a viable alternative in BS Zones I–II. Consistent outcome metrics and BS zonal stratification are crucial to refine surgical selection and optimize decision-making.

## Introduction

Gliomas of the insular region are relatively common within the spectrum of diffuse low-grade gliomas [1]. Although early studies characterized insular gliomas predominantly as low-grade tumors, more recent evidence indicates that up to 40% of these lesions may present as high-grade [2]. Furthermore, they represent a true challenge for oncological neurosurgery, given the region’s difficult surgical access and complex vascular anatomy [3–5]. Traditionally, this has been associated with a high rate of neurological complications in insular surgery, as more extensive resections - despite offering improved oncological outcomes - may lead to higher rates of neurological and functional deficits [4–6]. Consequently, due to the multiple factors that must be weighed when approaching insular gliomas, there is wide variation in management strategies across institutions, particularly regarding the choice of surgical approach [7].

Historically, the anatomical and vascular complexity of the insula has led many of its tumors to be considered unresectable. However, the pioneering work of Yasargil in the early 20th century identified specific growth patterns of certain insular tumors, suggesting their potential for surgical removal through dissection of the Sylvian fissure and thereby establishing the transsylvian (TS) approach [8]. A major advantage of this technique lies precisely in avoiding the frontal and temporal opercula, thus reducing the risk of functional impairment, especially in the dominant hemisphere. Nevertheless, with the advent of cortical stimulation and functional brain mapping, the transcortical (TC) approach has gained prominence, allowing for the creation of resection cavities within non-eloquent areas and thereby preserving both functional tissue and the transsylvian fissure with its associated vascular structures [9].

In this context, the Berger–Sanai (BS) classification, first published in 2010 [2], was introduced as a tool to support surgical decision-making. This classification divides the insula into four zones and serves to guide the assessment of resectability and the surgical management of insular gliomas [2, 9, 10]. Nonetheless, even with recent advances in the treatment of these tumors, the literature has not reached a consensus on which of the two main surgical approaches should be preferred, although a slight inclination toward the TC approach can be observed^7^.

Accordingly, the present study aims to compare the efficacy, safety, and prognostic factors associated with the two principal surgical approaches (TC and TS) to insular gliomas across the different BS zones, through a systematic review of the existing literature.

## Methods and materials

### Eligibility criteria

#### Inclusion

The selection of articles followed specific and strict criteria: adult patients (≥ 18 years) who underwent surgical resection of insular gliomas, regardless of WHO histological grade, provided that the lesion was predominantly centered in the insular lobe. Eligible interventions must have been performed using either a transcortical or a transsylvian approach, including recognized variants. Studies must stratify tumor location according to the BS classification (zones I–IV) and report both extent of resection and postoperative neurological deficits (immediate and/or delayed). Eligible designs include controlled observational studies, prospective or retrospective case series, cohort studies, and detailed case reports. No minimum follow-up duration will be required. Only peer-reviewed articles in English, published between January 1, 2010, and July 31, 2025, will be considered.

#### Exclusion

Duplicate records, studies with inadequate design (narrative reviews, meta-analyses, expert opinions), and abstracts or unpublished manuscripts will be excluded. Studies will also be excluded if they fail to report the outcomes of interest, if data are incomplete or aggregated, or if tumors are not classified anatomically according to BS. Gliomas predominantly extra-insular, publications in languages other than English, and studies outside the defined time frame will likewise be excluded.

### Search strategy and data collection

The literature search was conducted in PubMed/MEDLINE, Embase, Cochrane Library (including CENTRAL), and Google Scholar. The last search was performed on July 31, 2025. Controlled vocabulary and free-text terms related to “insular glioma,” “insula,” “glioma,” “insular cortex,” “transsylvian,” “transcortical,” “patient series,” and “series” were combined using Boolean operators. The search strategy was adapted to each database. Filters were applied to restrict results to adult humans, English-language publications, and publications from 2010 to 2025.

**Fig. 1:**
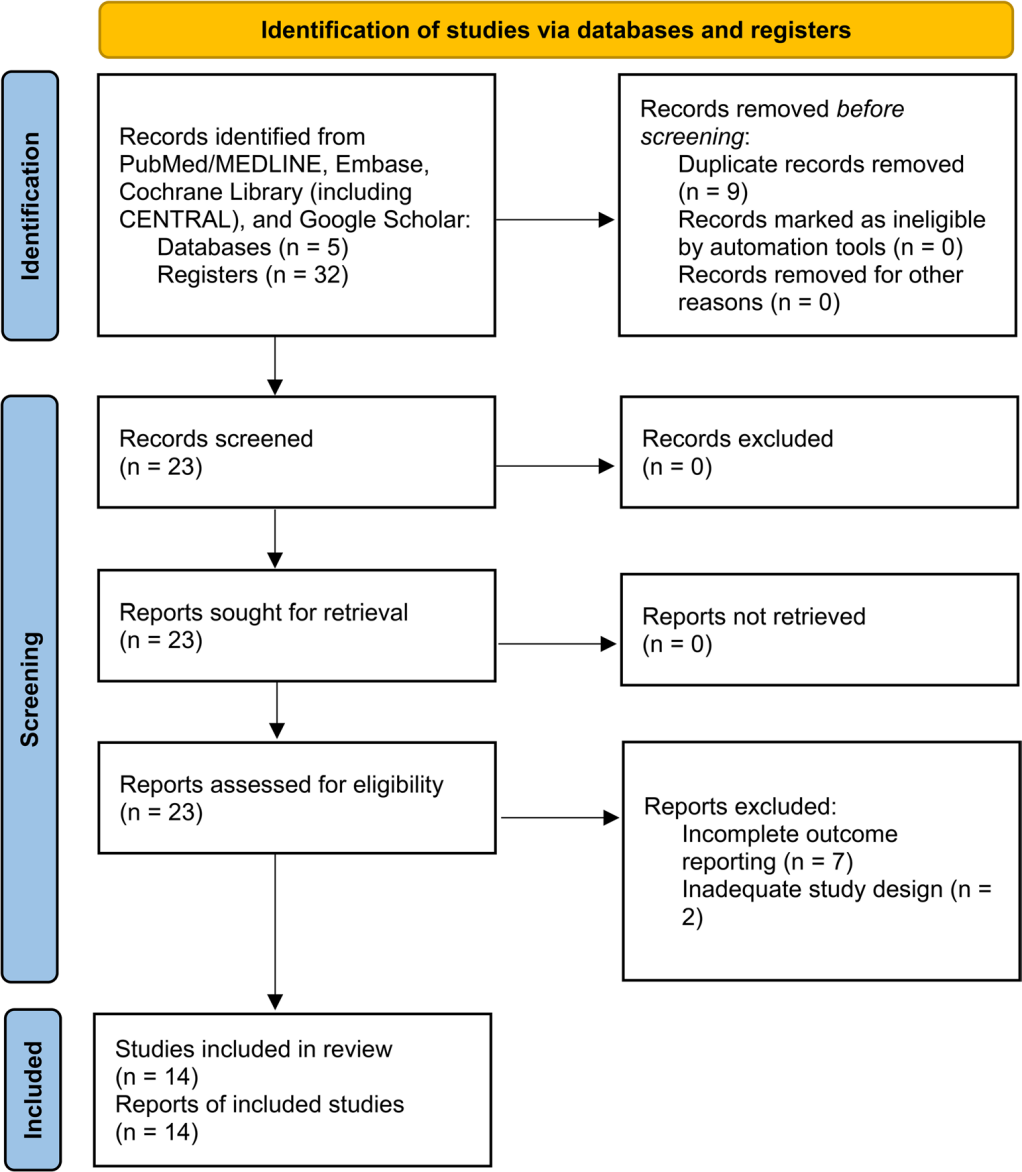
PRISMA flowchart

All records were exported to a reference manager, and duplicates were removed. Three reviewers independently screened titles and abstracts. Potentially eligible articles were assessed in full text. Discrepancies were resolved by consensus or arbitration by a fourth reviewer. In total, 32 records were identified; after removing 9 duplicates, 23 articles remained for screening. Of these, 9 were excluded for not meeting inclusion criteria, resulting in 14 full-text articles assessed. Finally, 14 studies were included in the qualitative synthesis, and, due to the lack of sufficient information, 2 were further excluded [11, 12], and 12 provided data for qualitative analysis. Reasons for exclusion at full-text review included absence of incomplete outcome reporting (7), and inadequate study design (2). The full study selection process is illustrated in the flowchart (Fig. 1).

Data extraction was performed independently using a standardized spreadsheet. Extracted variables included study data (year, country, design, period, sample size), patient/tumor information (age, sex, lateralization, WHO grade, dominance, volume, molecular markers when reported, BS zone), surgical approach (TC or TS, technical variants, mapping modalities, intraoperative imaging, DTI, neurophysiological monitoring), and outcomes. Extent of resection was recorded as volumetric percentage and harmonized across studies. Neurological deficits were classified as immediate (≤ 30 days) or delayed (> 30 days), with permanent deficits prioritized due to more consistent reporting. Secondary outcomes, when available, included operative time and postoperative ischemia.

### Quality assessment and risk of bias

Given the inclusion of case reports and case series in our systematic review, the methodological quality of these studies was evaluated using the Joanna Briggs Institute (JBI) Critical Appraisal Checklists. Case reports were assessed according to the JBI Case Report Checklist, which includes domains such as patient description, clinical history, diagnostic assessment, intervention, follow-up, outcomes, and key lessons. Case series were evaluated using the JBI Case Series Checklist, encompassing criteria including inclusion criteria, consecutive participant enrollment, standardized interventions, reliable outcome measurement, adequate follow-up, and appropriate descriptive statistics. Each item was rated as “Yes,” “No,” or “Unclear.” Discrepancies were resolved by consensus between two independent reviewers.

### Qualitative synthesis

Study characteristics, extent of resection, neurological outcomes, and ischemic rates were summarized descriptively. A quantitative analysis was not performed due to marked methodological heterogeneity and reporting inconsistencies across the included studies. Regarding EOR, data varied significantly: while a subset of studies utilized multiple categorical intervals, others reported only mean values with upper and lower bounds. Furthermore, neurological outcomes lacked standardization in both temporal classification and conceptual definitions. These discrepancies precluded a robust quantitative synthesis.

Whenever feasible, comparisons were made across BS zones (I–IV), intraoperative adjuncts, and histological grade. Sensitivity considerations included exclusion of studies with high risk of bias or isolated case reports. Data extraction was performed independently by reviewers using a standardized spreadsheet. Information collected included study characteristics (year, country, design, inclusion period, sample size), patient and tumor characteristics (age, sex, lateralization, WHO grade, hemispheric dominance, volume when available, BS zone), surgical details (transcortical or transsylvian approach and technical variants), and outcomes. Extent of resection was extracted as volumetric percentage and categorized when necessary, with data harmonized across study tables. Neurological deficits were classified as immediate or delayed according to articles definitions, prioritizing delayed ones (persisting at last follow-up) due to a noticeable standard of long-term outcomes documentation. Secondary outcomes, when available, included operative time and postoperative ischemia. Data from all included studies were manually extracted, and the review focused on qualitative synthesis of study characteristics, extent of resection, neurological outcomes, and seizure control. As this study involved analysis of previously published data, ethical approval was not required.

## Results

### Results of individual studies

14 studies fulfilled the eligibility criteria, encompassing 1,225 patients with predominantly insular gliomas. Of these, 235 underwent transsylvian resection and 990 were treated via a transcortical approach. The principal study features and outcomes are summarized in Table 1.

**Table 1:** Studies and its key characteristics, *excluded from qualitative analysis

	Key characteristics
Mamadaliev et al. (2025)	Reported a rare postoperative complication—olfactory hallucination—after insular glioma transcortical surgery.
Das et al. (2025)	Compared tumor resection rates and complication profiles of transsylvian versus transcortical approaches for insular gliomas.
Noll et al. (2024)*	Diagnosed insular glioma patients underwent awake resection (64% transsylvian, 36% transcortical), focusing on post-operative outcomes.
Nery et al. (2024)	Two case reports comparing transcortical and transsylvian approaches.
Panigrahi et al. (2021)	Retrospective analysis of consecutive insular glioma resections (2013–2016), focusing on post-operative neurological deficits and mortality up to three months.
Rossi et al. (2021)	Five-year retrospective study of giant insular gliomas resected via transcortical approach with brain mapping, analyzing extent of resection and postoperative outcomes.
D. Pitskhelauri (2021)	From 2012 to 2017, 79 patients underwent transsylvian resection of insular gliomas, with extent of resection assessed via MRI within 48 h postoperatively.
Li et al. (2020)	Analyzed patients undergoing their first transcortical insular glioma resection at their institute between March 2011 and July 2019.
Przybylowski et al. (2019)	100 consecutive insular glioma resections were analyzed for extent of resection and postoperative ischemia, with logistic regression identifying predictors of neurological morbidity.
Mandonnet (2019)	Retrospective review of insular glioma resections via transopercular approach; assessed extent of resection, postoperative ischemia, and neurological, neuropsychological, and professional outcomes.
Hameed et al. (2019)	Analyzed 255 consecutive transcortical insular glioma resections, assessing tumor pathology, location, extent of resection, neurological outcomes, and overall survival.
Chen et al. (2017)*	Five-year retrospective study of giant insular gliomas resected via transcortical approach with brain mapping, evaluating factors affecting extent of resection and postoperative neurological and neuropsychological outcomes.
Eseonu et al. (2017)	Analyzed 74 initial insular glioma resections (2006–2016) to assess the prognostic impact of volumetric extent of resection and molecular markers on survival for low- and high-grade tumors.
Sanai et al. (2010)	Adult patients with WHO Grade II–IV insular gliomas were analyzed for tumor location (BS Zones I–IV), interobserver classification variability, and tumor volumes via FLAIR and contrast-enhanced T1 MRI.

### Classification attempts and its synthesis

Comparative synthesis was hindered by marked heterogeneity in outcome reporting. EOR was inconsistently defined - alternating between gross total, subtotal, and volumetric measures - and frequently reported without BS zone–specific stratification [7]. In addition, some series that initially separated transcortical and transsylvian cohorts later merged their data, further impairing direct comparison [13].

Definitions of postoperative neurological deficits were likewise inconsistent: some authors classified deficits persisting beyond 1 month as “late” [14], others adopted a 3-month cutoff [15]. Such disparity complicated uniform endpoint construction. Moreover, outcomes were influenced by operator experience, with less seasoned surgeons exhibiting higher morbidity [16].

Overall, classification and reporting of insular glioma resections remained markedly heterogeneous, driven by methodological disparities and institution-specific practices. This variability limits the strength of comparative inferences and emphasizes the need for standardized reporting frameworks in future studies (Table 2).

**Table 2:**
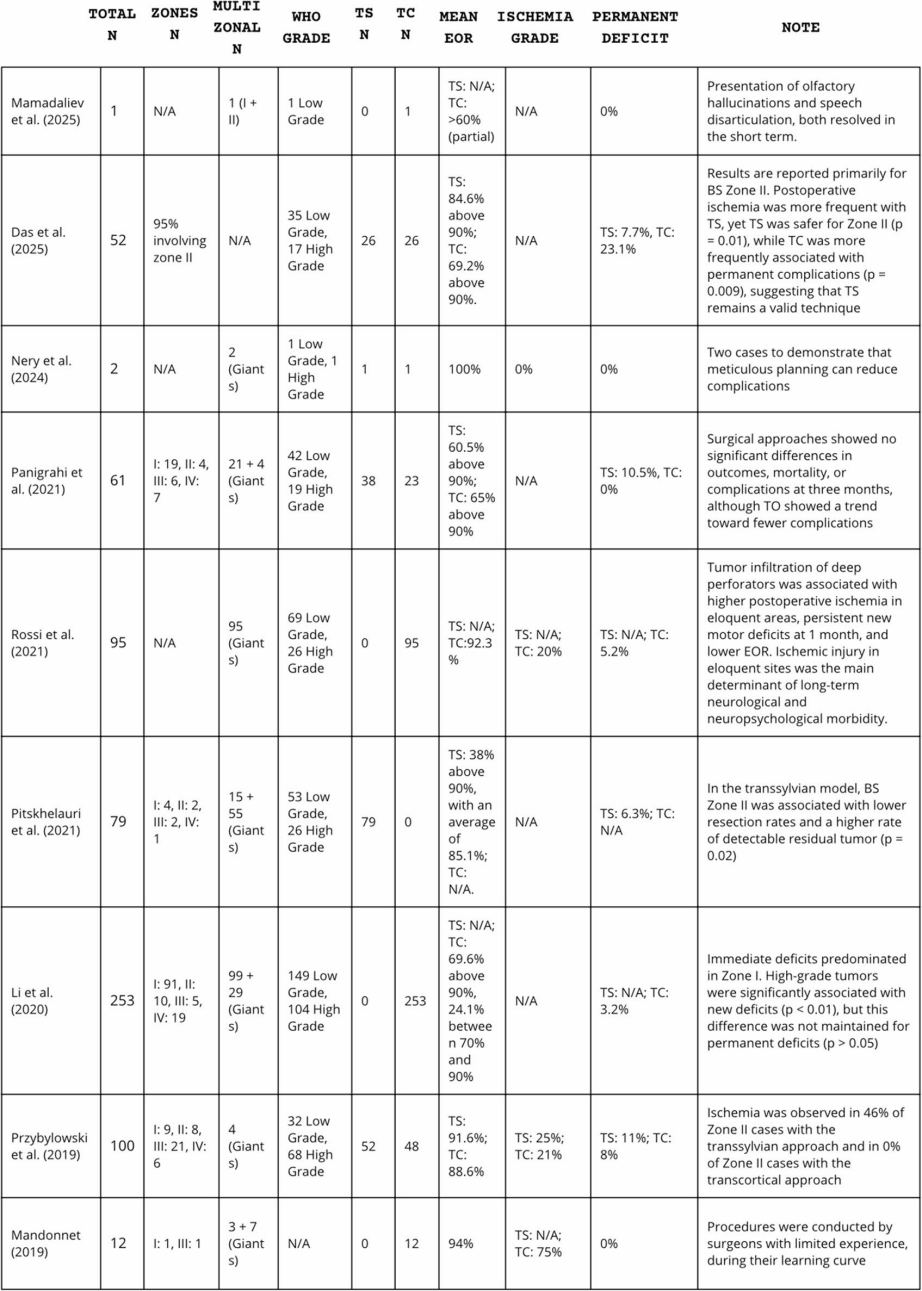

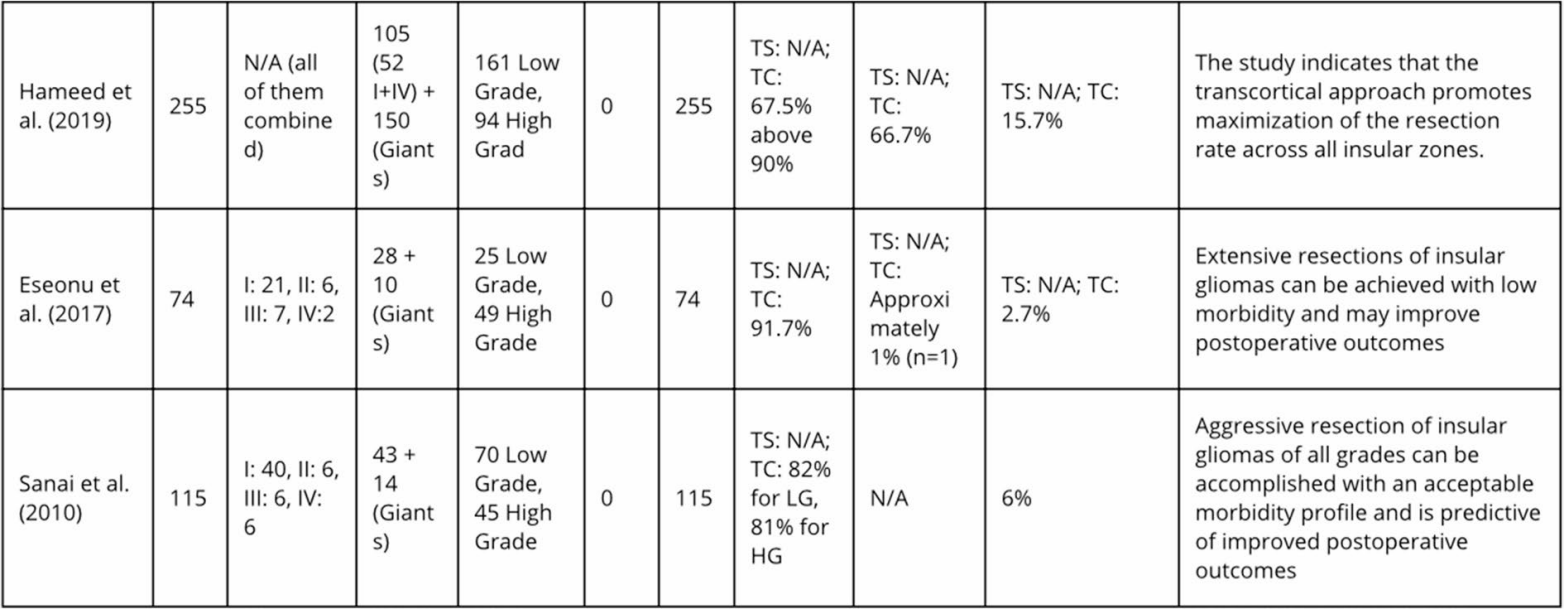
Data extraction

### Risk of bias

The methodological quality of the included studies ranged from moderate to high. Large institutional series [2, 17–19] demonstrated clearly defined inclusion criteria, reliable diagnostic confirmation, and standardized volumetric assessment of the extent of resection (EOR), collectively indicating a low risk of bias. Their principal limitation was the minimal reporting of demographic characteristics, which constrains external validity.

Rossi [14] provided a substantial single-center cohort with a strong focus on functional and neuropsychological outcomes, methodologically robust, though oriented toward endpoints distinct from survival. Mandonnet [16] offered valuable technical insight into transcortical resections but was limited by its small sample. Das [7] strengthened internal validity through propensity score matching, yielding the most analytically rigorous comparison between TS and TC approaches despite a modest cohort size.

Panigrahi [15] emphasized the impact of intraoperative adjuncts but relied on subjective EOR estimates and short follow-up. Conversely, Przybylowski [20] employed precise volumetry and systematic magnetic ressonance, showing reduced ischemic risk with the transcortical route in Zone II tumors and identifying high-grade histology as the primary driver of permanent morbidity.

Case reports [21, 22] contributed detailed technical observations but inherently lacked consecutive inclusion, demographic characterization, and long-term follow-up.

Taken together, large case series provide the most reliable evidence on EOR, morbidity, and survival, while smaller series and case reports add technical and functional nuance. The convergent literature underscores the need to tailor surgical strategy to tumor zone, histological grade, individual anatomy, and the judicious use of intraoperative adjuncts to mitigate morbidity and preserve function.

### Extent of Resection (EOR)

#### Transcortical versus Transsylvian approach

The EOR was heterogeneous across the included studies, varying both in patient proportion and resection rates. Only one study reported complete tumor resection with no difference between surgical approaches [22].

Studies restricted to the TC approach consistently demonstrated high EORs. Sanai [2] reported EORs of 82% in low-grade and 81% in high-grade tumors, while Eseonu [17] and Mandonnet [16] observed mean EORs of 91.7% and 94%, noting that the latter series involved surgeons still in their learning phase. More recent reports likewise achieved EOR > 90% in most cases [14, 18, 19]. In contrast, the only series focused exclusively on the TS approach reported EOR > 90% in just 38% of patients, with particularly high residual volumes in BS Zone II lesions [23].

Nevertheless, comparative studies showed higher EORs in the TS group. Chen [11] observed differences between TC and TS but did not report explicit mean values. Przybylowski [20] reported lower EOR in the TC group (88.6%) compared to TS (91.6%), while Das [7] observed 84.6% for TS versus 69.2% for TC. Conversely, Panigrahi [15] reported a slight advantage for TC when considering the proportion of patients achieving > 90% resection. Overall, TC was the most frequently employed approach and was associated, in most studies, with higher EOR.

#### Berger-Sanai Zones

Across the analyzed studies, BS Zone I consistently yielded the highest EOR among insular regions. Sanai [2], whose cohort showed a predominance of Zone I lesions (*n* = 40), reported mean EORs of 82% for low-grade and 81% for high-grade gliomas using transcortical routes. Przybylowski [20] likewise demonstrated high resectability, with EORs of 91.6% via the TS approach and 88.6% via the TC approach, underscoring the favorable microsurgical anatomy of this anterior compartment. Pitskhelauri [23] further documented an average EOR of roughly 85% through a purely TS route, reinforcing the view that Zone I affords superior exposure with comparatively limited eloquence-related risk.

Zone II tumors pose a distinct technical challenge. Das [7], analyzing 52 cases, reported EOR > 90% in 84.6% of TS resections versus 69.2% of TC procedures; despite a higher ischemia rate, the TS approach showed greater overall efficacy for this compartment (*p* = 0.01). In contrast, Pitskhelauri [23] associated Zone II with lower gross-total resection rates and more frequent radiologically evident residuals (*p* = 0.02), highlighting the complex vascular and subinsular anatomy that can limit the effectiveness of the transsylvian corridor. Przybylowski [20] additionally reported ischemic changes in 46% of TS resections for Zone II lesions, absent in their TC cases, underscoring the trade-off between exposure and vascular safety. Subsequently, Przybylowski [24] suggested that the TC route offers superior visualization for posterior regions (Zones II and III), enabling safer and more complete resections. Taken together, these studies reveal substantive discordance, although the findings of Das [7] carry particular weight given their larger Zone II cohort relative to those other ones.

Przybylowski [20] reported the largest cohort of Zone III tumors, comprising 21 cases with EORs above 85%, albeit with slightly reduced completeness compared with Zones I-II. This region, being the deepest and most posterior-inferior quadrant of the insula, is consistently regarded as the least amenable to complete resection. Their data further indicated that the TC route affords more direct access than the TS approach in this compartment.

Li [18] reported EORs of approximately 85% for anterior lesions, decreasing to 45.8% for posterior variants. Their cohort, which included the largest proportion of Zone IV (anterior-inferior) tumors, and employed exclusively TC access, achieved an overall EOR of 89%, second only to that observed in Zone I. Owing to its position along the sylvian line, Zone IV can be exposed through either TC or TS corridors. Panigrahi [15] confirmed the feasibility of both approaches, while noting that the greater retraction required for the TS route may confer a higher risk of tissue injury.

### Permanent neurological deficits

#### Transcortical versus Transsylvian approach

Permanent neurological deficits were not uncommon, but their incidence varied widely across studies. In studies evaluating the TC approach alone, permanent deficit rates were relatively low: Eseonu [17] reported 2.7%, Sanai [2] 6%, Rossi [14] 5.2%, and Li [18] 3.2%. Hameed [19], despite limited data, also documented low permanent morbidity. Similarly, a study focusing exclusively on the TS approach reported 6.3% permanent deficits [23].

Comparative studies showed greater heterogeneity. Przybylowski [20] reported 11% in TS versus 8% in TC. Panigrahi [15] observed 10.5% in TS and 0% in TC. Das [7] reported 7.7% in TS versus 23.1% in TC, with a statistically significant association between TC and higher risk of permanent complications (*p* = 0.009). Other studies either found no differences between approaches or did not report deficits [11, 22].

Overall, both TC and TS approaches can be performed with low rates of permanent neurological deficits in experienced centers. However, variability may be influenced by tumor grade, and surgical experience, with some studies suggesting higher morbidity in TC, particularly during the learning curve or in the absence of adequate functional monitoring.

The specific deficit characteristics and outcomes were systematically extracted to provide a more granular view of the surgical landscape (Table 3). Regarding surgery, operative time was satisfactorily reported in only one study [14].

**Table 3:**
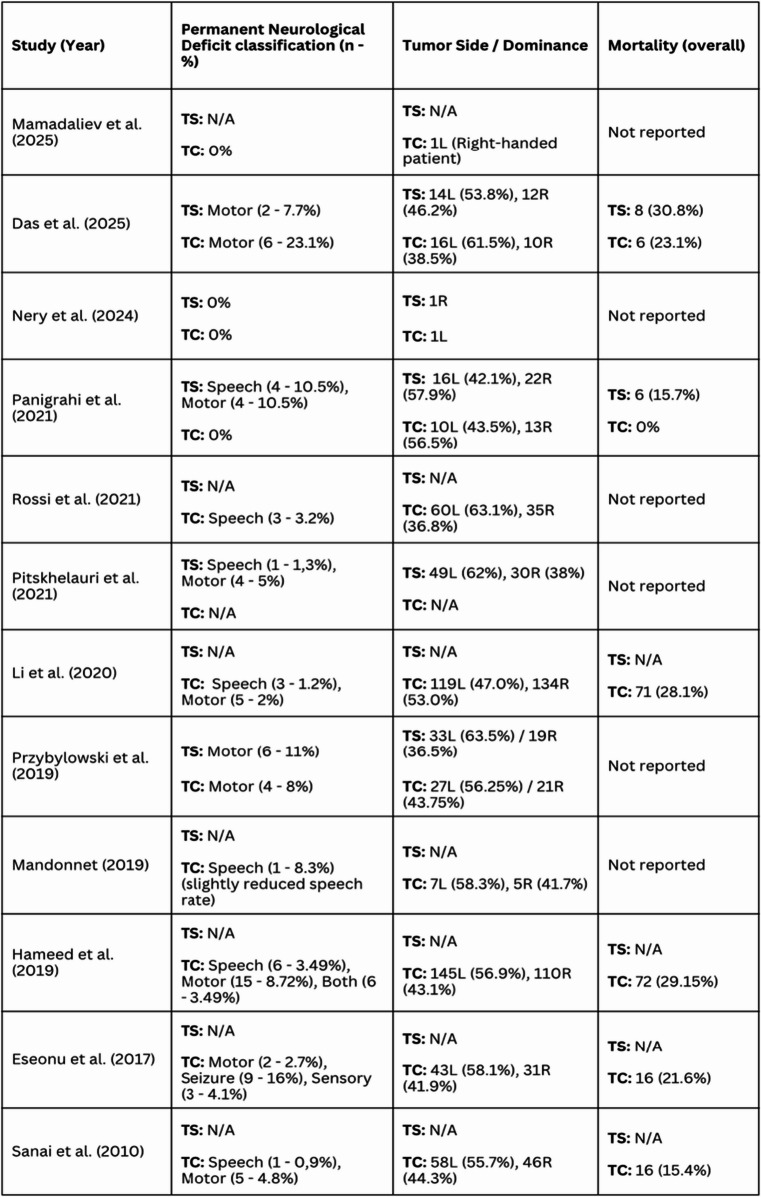
Classification of neurological deficits and overall mortality across included studies

Abbreviations: *TC* Transcortical; *TS* Transsylvian; *R* Right, *L* Left, *N/A* Not Applicable

#### Berger-Sanai Zones

Zone I consistently demonstrates the lowest absolute rates of permanent neurological deficits among the reviewed series. Przybylowski[20], despite including a substantially higher proportion of high-grade gliomas, reported only modest rates of lasting deficits (TS 11% vs. TC 8% overall), with no indication that Zone I disproportionately contributes to permanent morbidity. Likewise, Li [18] noted that although transient postoperative impairments were relatively frequent in this anterior-superior region, the vast majority resolved over time, indicating that aggressive resections in Zone I are both technically feasible and functionally safe when guided by meticulous intraoperative mapping.

Zone II exhibits a more complex and nuanced profile. Although the EOR can be remarkably high, exceeding 90% in TS series [7], this territory is consistently associated with an elevated risk of postoperative ischemia and consequent functional sequelae. Das [7], whose cohort consisted almost entirely of Zone II tumors (95%), reported a substantial difference in permanent deficits between approaches (TS 7.7% vs. TC 23.1%). Also, Przybylowski [20] observed ischemic changes in 46% of Zone II resections performed via the TS route, compared with 0% in TC cases, although these ischemic events did not necessarily culminate in higher rates of permanent neurological morbidity (TS 11% vs. TC 8%). Pitskhelauri [23] additionally noted a higher incidence of residual tumor following transsylvian resections in this zone, highlighting the restrictive influence of its intricate vascular architecture on both completeness and surgical safety. Collectively, these findings underscore that Zone II embodies a critical trade-off between maximal resectability and ischemia-mediated functional risk, with outcomes heavily dependent on microsurgical technique and operator expertise.

**Fig. 2:**
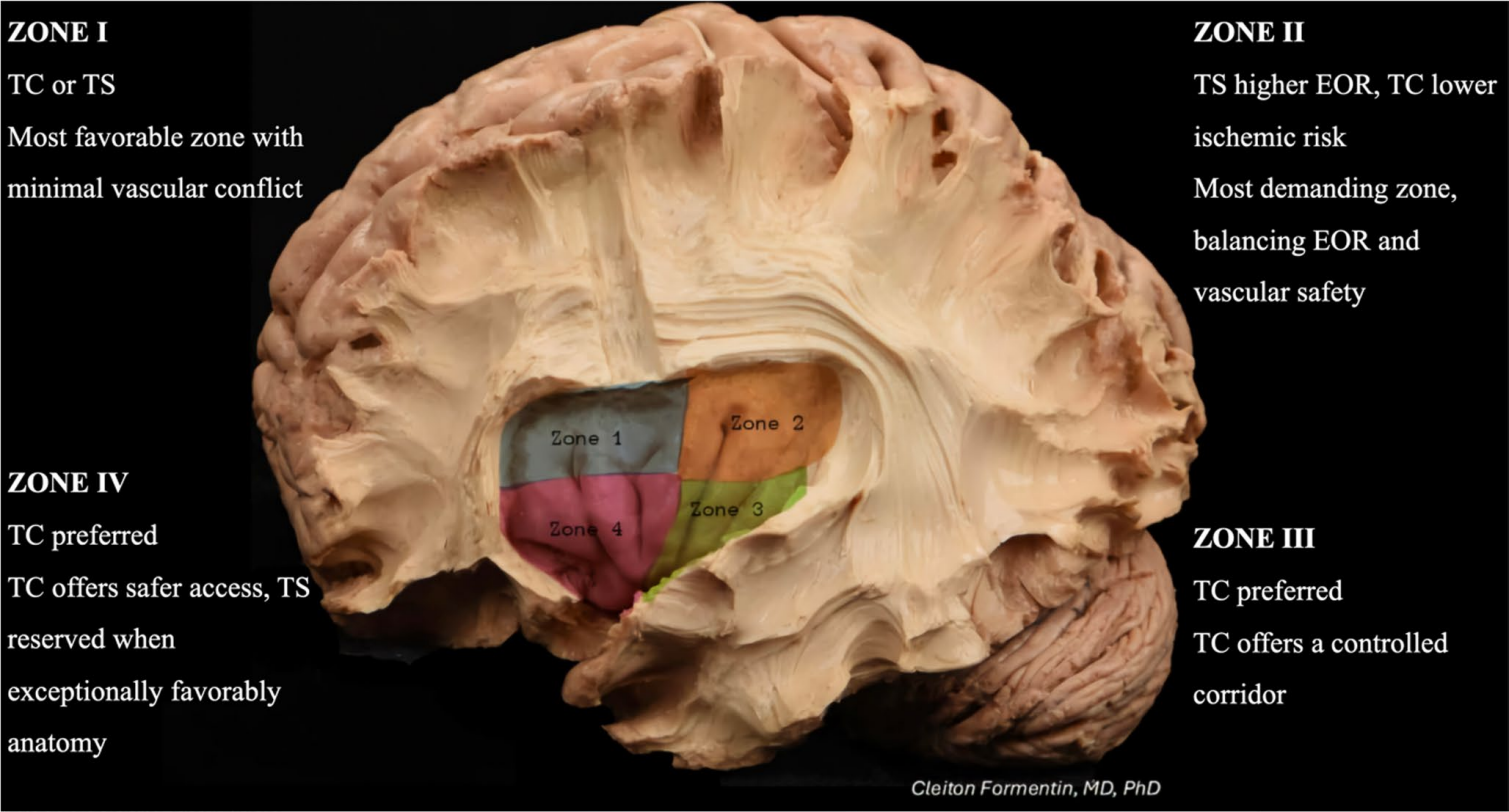
Synthesis of results across Berger-Sanai Zones

Zones III and IV were less extensively characterized in most series with regard to permanent neurological morbidity. In the dataset by Przybylowski [20], which included a substantial number of cases in Zone III (*n* = 21) and Zone IV (*n* = 6), permanent deficits were reported at rates of TS 11% and TC 8%, but without distinct zonal stratification. These observations suggest that these zones are comparatively underrepresented in the literature and, by extension, may hold a more modest clinical relevance than the other insular regions (Fig. 2).

## Discussion

### Assessment of data

Synthesis across studies was limited by substantial heterogeneity in reporting EOR and neurological outcomes. EOR was variably expressed as gross total resection, subtotal resection, or volumetric percentage, and many studies aggregated results across multiple BS zones, hindering zone-specific comparison [7, 21]. In some cases, initial differentiation between TC and TS approaches was later combined, further limiting direct analysis [13].

Definitions of permanent neurological deficits were inconsistent, ranging from 1 to 3-month thresholds [14, 15]. This methodological divergence directly influences reported morbidity rates. The disparity in follow-up periods, the lack of a standardized benchmark to determine the presence of deficits, and other inconsistencies across the records contribute to reported rates ranging from 0% to 23%. These figures reflect not only differences in surgeon experience, intraoperative monitoring, and tumor complexity, but also fundamental methodological discrepancies [14, 16].

These inconsistencies limit firm comparative conclusions and highlight the need for standardized reporting, including clear EOR metrics, uniform neurological endpoints, and systematic stratification by BS zone, tumor grade, and surgical experience. 

### EOR and neurological deficits

The present synthesis confirms that both the TC and TS approaches enable substantial resection of insular gliomas, yet the EOR and neurological morbidity exhibit significant heterogeneity across studies. These differences appear to depend not only on tumor volume, histology, and surgical experience, but crucially on the spatial distribution of the lesion according to the BS zonal classification.

 Across the 14 included studies encompassing 1,225 patients, the TC approach was more frequently employed, often resulting in higher EOR. Rossi [14] reported a mean EOR of 92.3% for TC in a cohort of 95 patients, while Li [18] achieved resections exceeding 90% in 69.6% of TC cases. In contrast, TS-exclusive series, such as Pitskhelauri [23], achieved ≥ 90% EOR in only 38% of patients, most of which were concentrated in BS zone II. Comparative studies produced heterogeneous findings: Przybylowski [20] observed slightly higher EOR with TS (91.6%) versus TC (88.6%), whereas Das [7] reported EOR above 90% in 84.6% of TS cases compared to 69.2% for TC. Panigrahi [15] noted a marginal advantage for TC (65%) over TS (60.5%). These inconsistencies likely reflect differences in tumor zonal distribution and the relative dominance of anterior (Zones I–IV) versus posterior (Zones II–III) lesions within each cohort, reinforcing that surgical strategy cannot be interpreted independently of anatomical context.

The variability identified in deficit reporting, including in follow-up duration and the lack of detailed etiology for overall mortality, underscore the diverse follow-up protocols. Operative time was exclusively reported by Rossi [14], highlightinag an intrinsic disparity in how different surgeons prioritize and interpret clinical metrics. Such heterogeneity explains the wide range of reported outcomes and confirms the necessity of a qualitative approach to synthesize the current evidence.

 When analyzed by BS zones, Zone I consistently demonstrated the most favorable balance between EOR and functional preservation. Przybylowski [20] reported long-term deficits in this zone about TS 11% vs. TC 8%, despite a predominance of high-grade lesions. Li [18] similarly observed that early postoperative deficits in the anterior-superior region were largely transient, suggesting that aggressive resections within Zone I are feasible and functionally safe under mapping guidance. Thus, no notable differences were identified regarding the surgical technique to be employed.

Zone II, however, displayed a more ambiguous profile. Although several TS series achieved EOR exceeding 90% [7], this region was recurrently associated with postoperative ischemia and higher risk of permanent morbidity. Das [7] reported 7.7% permanent deficits for TS versus 23.1% for TC in predominantly Zone II tumors, while Przybylowski [20] documented ischemic changes in 46% of TS cases within this zone (compared to 0% for TC), although most were not clinically permanent. The intricate vascular configuration and proximity to lenticulostriate arteries in Zone II likely constrain both resection completeness and functional safety, requiring exceptional microsurgical precision.

Zones III and IV, which occupy the inferior insular sectors, did not exhibit major zonal differences in EOR but were consistently associated with greater technical difficulty due to their proximity to deep eloquent pathways. In this context, the transsylvian route remains acceptable primarily for Zones I, II, and the sylvian-aligned portion of Zone IV, whereas Zone III has shown a shift toward preferential use of the transcortical approach.

Permanent neurological deficits were generally low in specialized centers, but rates varied according to both approach and BS zone. TC-only series reported permanent deficits ranging from 2.7% [17] to 6% [2], while TS-only series showed 6.3% [23]. In direct comparisons, outcomes diverged considerably: Das [7] found significantly higher morbidity with TC (23.1%) than TS (7.7%), whereas Przybylowski [20] reported the inverse trend (TS 11% vs. TC 8%), and Panigrahi [15] noted 0% versus 10.5%, respectively. The postoperative ischemia, particularly in eloquent zones, emerged as a determinant of long-term neurological outcome, with TC-associated deficits more frequent in settings of limited intraoperative mapping or early learning curve.

Furthermore, it is essential to acknowledge that the BS classification is a strictly topographical framework. As noted in our analysis, this system facilitates surgical planning by dividing the insula into anatomical quadrants but does not incorporate the biological or morphological heterogeneity of the tumors. Clinical behavior and resectability are heavily influenced by whether a lesion is low-grade or high-grade, its growth pattern, and its radiological appearance (contrast-enhancing vs. non-enhancing). For example, a diffuse, non-enhancing low-grade glioma in a given insular zone may lack clear surgical planes and limit gross total resection, whereas a more focal high-grade lesion in the same location may present more defined operative boundaries despite its greater biological aggressiveness.

In summary, both TC and TS approaches can be safely implemented in experienced centers, achieving high EOR while maintaining low rates of permanent deficits. However, the choice of surgical corridor should be guided by tumor growth pattern, BS zone, anticipated functional risk, and surgeon expertise, highlighting the need for individualized surgical planning and careful integration of functional mapping techniques, rather than relying solely on the nominal surgical route. 

### Limitations

The standardization of data across the included studies was particularly challenging. Significant heterogeneity persisted in terms of surgical experience, methodological design, classification of neurological deficits, and reporting of extent of resection. In several instances, we had to rely on the data as originally reported, despite suboptimal methodological rigor. These issues, already detailed in the risk of bias assessment, should be considered when interpreting the results of this systematic review.

## Conclusion

 This systematic review of 14 studies including 1,225 patients, demonstrates that both TC and TS approaches achieve high extents of resection, with the potential for low overall rates of permanent neurological deficits. Crucially, outcomes are strongly influenced by tumor location within the BS framework. Zone I consistently allows maximal resection with minimal functional risk, while Zone II presents a critical trade-off between high resectability and increased ischemia-mediated morbidity, emphasizing the need for meticulous microsurgical technique. While the former shows favorable outcomes for both TC and TS, the latter appears to be better managed via TS.

Zones III and IV show an increasing preference for transcortical access, given its superior visualization and operative control. Although Zone IV can be reached through a transsylvian corridor, this route carries a higher risk profile. These observations reinforce the necessity of BS zonal stratification in surgical planning and highlight the need for future studies to report EOR and functional outcomes by zone to strengthen evidence-based decision-making in insular glioma surgery.

## Data Availability

No datasets were generated or analysed during the current study.
